# Age-related alterations in functional and structural networks in the brain in macaque monkeys

**DOI:** 10.3389/fnana.2025.1495735

**Published:** 2025-03-25

**Authors:** Kazuya Ouchi, Shinya Yamamoto, Makoto Obara, Yasuko Sugase-Miyamoto, Tomokazu Tsurugizawa

**Affiliations:** ^1^Human Informatics and Interaction Research Institute, National Institute of Advanced Industrial Science and Technology, Tsukuba, Japan; ^2^Faculty of Engineering, Information and Systems, University of Tsukuba, Tsukuba, Japan; ^3^Graduate School of Comprehensive Human Sciences, University of Tsukuba, Tsukuba, Japan; ^4^Philips Japan Ltd, Tokyo, Japan; ^5^Université du Québec à Trois-Rivières, Trois-Rivières, QC, Canada

**Keywords:** macaque monkey, functional connectivity, structural connectivity, network, MRI

## Abstract

Resting-state networks (RSNs) have been used as biomarkers of brain diseases and cognitive performance. However, age-related changes in the RSNs of macaques, a representative animal model, are still not fully understood. In this study, we measured the RSNs in macaques aged 3–20 years and investigated the age-related changes from both functional and structural perspectives. The proportion of structural connectivity in the RSNs relative to the total fibers in the whole brain significantly decreased in aged macaques, whereas functional connectivity showed an increasing trend with age. Additionally, the amplitude of low-frequency fluctuations tended to increase with age, indicating that resting-state neural activity may be more active in the RSNs may increase with age. These results indicate that structural and functional alterations in typical RSNs are age-dependent and can be a marker of aging in the macaque’s brain.

## 1 Introduction

Resting-state networks (RSNs) in the brain that reflect cognitive function are dependent on aging in humans (Goelman et al., 2024). Importantly, macaque monkeys have RSNs homologous to those in humans (Hutchison et al., 2011). The previous study investigating age-related differences in RSNs among macaques, ranging in age from 1 to 15 years, reported that the RSNs of monkeys were not widely changed (Rao et al., 2021). However, that study did not investigate change of age in term of structural connectivity (SC), Functional connectivity (FC), amplitude of low-frequency fluctuations (ALFF), global correlation, and local correlation in RSNs. Another study that surveyed structural T1-weighted MRI scans of 66 macaques (aged 5–31 years) reported enlargement of the lateral ventricles and smaller volumes in the frontal cortex, caudate, putamen, hypothalamus, and thalamus in older macaques compared to their younger counterparts (Dash et al., 2023). The regional cerebral blood flow and the regional metabolic rate of glucose in aged macaques were lower than those in young macaques (Noda et al., 2002). Age-related changes in metabolic rate of glucose in specific brain regions, such as the hippocampus and prefrontal cortex, are associated with cognitive performance (Eberling et al., 1997; Upright and Baxter, 2021), indicating that changes in the metabolism in these regions are associated with cognitive performance in aged people. As neuronal and astrocyte activity require energy metabolism, altered energy metabolism should be relevant to the blood oxygenation level-dependent (BOLD) signal, a signal source of fMRI regulated by neuronal and astrocyte activity. The normal aging process in humans is accompanied by a restructuring of the FC networks, characterized by a shift from more segregated to more integrated brain networks, which has been found to be important for changes in our cognitive performance. The default mode network activity during resting state reflects internal mental processes occurring without external stimuli. This activity correlates with performance on specific cognitive functions in the cognitive composite score, which includes paired associates immediate recall, logical memory delayed recall, confrontational word retrieval, and digit-symbol substitution (Oishi et al., 2022). In addition to the alterations in FC with age, human MRI studies have revealed age-dependent alterations in SC (Stumme et al., 2022). The previous study has shown that brain volume decreases with age (Dash et al., 2023). However, the relationships among SC, FC, and age in macaques have not yet been fully investigated. In addition to FC and SC, some indices were calculated from the spontaneous fluctuations of functional magnetic resonance imaging (fMRI) signal changes depending on age. The ALFF, global correlation, and local correlation measured during RSN have also been associated with age-dependent metabolism in humans (Bernier et al., 2017; Tomasi et al., 2013). The ALFF is an index calculated from the square root of the power spectrum in the frequency range, generally between 0.008 and 0.1 Hz, and it has been validated as a useful biomarker of the brain’s physiological state (Yang et al., 2007). A previous study reported that ALFF increases with age in humans (Zhang et al., 2021) and increase of ALFF in the left cerebellar posterior lobe and left superior parietal gyrus are correlated with behavioral performance, such as delayed processing speed in trail making test (Fan et al., 2022). Global correlation analyses have indicated correlations between the signal fluctuation at each voxel and that averaged in the whole brain, whereas local correlation has indicated the synchronization between a specific voxel and neighboring voxels. These have been utilized as indices to demonstrate the coordination between neurons (Deshpande et al., 2009). Therefore, it can be considered an age-dependent index similar to ALFF. In contrast to humans, whether these indices change with age in macaques remains unclear. We therefore hypothesized that age-related changes in these indices of macaques were similar to those in humans. This study aimed to investigate the relationship between brain function and structure and age in macaques. We investigated age-related changes in SC, FC, ALFF, global correlation and local correlation within the RSNs in macaques using regression analysis. Therefore, we performed a regression analysis between age and brain structure or function.

## 2 Materials and methods

### 2.1 Subjects

This study used seven macaques (Table 1). The monkeys were housed in individual primate cages under controlled conditions of humidity, light and temperature. A standard diet and water were provided daily. All experiments were approved by the Ethics Committee of the National Institute of Advanced Industrial Science and Technology and carried out in accordance with the Guide for the Care and Use of Nonhuman primates in Neuroscience Research (The Japan Neuroscience Society).^1^

**Table 1:** Age, weight, sex of all macaque subjects.

Group	Subject number	Age (years)	Weight (kg)	Sex
Young	1	6	12.8	Male
	2	7	13.4	Male
	3	7	10.2	Male
	4	8	10.1	Male
	5	3	4.0	Female
	Mean ± std	6.2 ± 1.7	10.1 ± 3.3	
Old	6	16	11.6	Female
	7	20	9.8	Female
	Mean ± std	18 ± 2.0	10.7 ± 0.9	

Std means the standard deviation.

### 2.2 MRI acquisition

All MRI experiments were performed using a Philips Ingenia 3 T MRI system with an 8-channel brain array RF coil (Rapid biomedical, Germany). Macaques were anesthetized using a combination of medetomidine (0.05 mg/kg), midazolam (0.3 mg/kg), and ketamine (0.4 mg/kg) during MRI scanning, which was injected intramuscularly 30 min before the start of the survey scan. Diffusion-weighted imaging (DWI) was performed using a diffusion-weighted spin-echo echo planner imaging (EPI) sequence with the following parameters: repetition time (TR)/echo time (TE) = 20,000/93 ms, voxel size = 1.2 × 1.2 × 1.2 mm^3^ /voxel, matrix size = 112 × 112, 44 slices, b-value = 0, 1,000, and 3,000 s/mm^2^, and 64 directions. Resting-state functional MRI images were acquired using a gradient-echo EPI sequence with the following parameters: TR/TE = 2,000/30 ms, flip angle = 80°, voxel size = 1.3 × 1.3 × 1.3 mm^3^ / voxel, matrix size = 80 × 80, 35 slices, and a total of 10 min (300 volumes). The T1-weighted image was acquired for the registration in image in preprocessing, using a magnetization-prepared rapid gradient echo (MPRAGE) sequence with the following parameters: TR/TE = 13/5.8 ms, voxel size = 0.6 × 0.6 × 0.6 mm^3^/voxel, and matrix size = 200 × 250 × 167.

### 2.3 Structural connectivity (SC)

Preprocessing of the DWI data, including denoising, distortion correction, eddy current correction, motion correction, and B1 field inhomogeneity correction, was performed using the recommended pipeline provided in MRtrix3 version 3.0.4 (Tournier et al., 2019). Non-brain tissues were removed using FSL software version 6.0.7.2 (University of Oxford, United Kingdom).^2^ Then, we registered a template image and regions of interest (ROIs) to each subject’s DWI image using rigid, affine, and deformable B-spline SyN transformations. This registration process was performed using ANTs version 2.5.0 (Avants et al., 2009). The National Institute of Mental Health Macaque Template (NMT) (Jung et al., 2021; Seidlitz et al., 2018) and Cortical Hierarchy of Resus Macaques (CHARM) (Jung et al., 2021; Reveley et al., 2017) were used as template image and ROIs (Ouchi et al., 2024). Fiber tracking was performed for each macaque using “tckgen” function in MRtrix3. The number of selected streamlines was set to 100 million, and other tracking parameters were set to their default values. The seed region was dynamically determined using the “seed_dynamic” option with the SIFT model. Additionally, by using the “-act,” “-backtrack,” and “-crop_at_gmwmi” options, only white matter was tracked, poor structural terminations were allowed to retrack, and the streamline endpoints were cropped more precisely as they crossed the GM-WM interface. The streamlines were optimized with per-streamline cross-section multipliers using the “tcksift2” function. The SC was assigned as the absolute number of streamlines connecting each pair of ROIs using the “tck2connectome” function in MRtrix3 to create each subject’s SC matrix. The SC matrix is a symmetric matrix where each row and column correspond to ROI, and each element indicates the neural fibers connecting each pair of ROIs. The proportion of SC was calculated as the number of fibers connecting each RSN relative to the total number of fibers in the whole brain.

### 2.4 Functional connectivity (FC)

Statistical parametric mapping SPM12 software (Welcome Trust Center for Neuroimaging, London, United Kingdom) was used to preprocess the fMRI data. The slice timing was used to correct the gap of the acquisition timing in each slice in 2D-gradient echo EPI. The realignment was used for the spatial correction of the brain in each volume. The same template image used for the SC analysis was applied for the old normalization, and the voxel size of the subject’s image was employed. For smoothing, FWHM was set to twice the voxel size. The processed data were then processed using CONN toolbox version conn22a (Liu and Falahpour, 2020). Signals related to the six affine parameters of head motion and signals within the white matter and cerebrospinal fluid were regressed-out. The residue of the fMRI signals was then detrended, and slow periodic fluctuations were extracted using a bandpass filter (0.008–0.09 Hz) through a discrete cosine transform (Hallquist et al., 2013). The same ROIs as in the SC analysis were used to calculate the FC. FC represents the temporal correlation of the BOLD signal across the entire time series data (one session of 10 min recording) between each ROI, measured during approximately 10 min of resting-state brain activity, which is typical in humans (Oldehinkel et al., 2022) and macaques (Hindriks et al., 2016). We created FC matrix for each individual. FC matrix is a symmetric matrix where each row and column correspond to an ROI, and each component of the FC matrix was displayed as a Fisher-transformed bivariate correlation coefficient between the BOLD time series of a pair of ROIs.

### 2.5 Individual similarity of SC and FC

Similarities among participants were calculated using a custom-made program in MATLAB R2023a (MathWorks, Massachusetts, United States). For each subject, the upper triangular matrix of SC and FC were converted into one-dimensional vectors, which were then used as the feature vectors representing the subject’s SC and FC (Ouchi et al., 2024). Pearson’s correlation coefficients for SC and FC were calculated using feature vectors as the similarity between pairs across all individuals (Hyon et al., 2020). Then, a correlation matrix was created for both SC and FC, where rows and columns represent subjects, and each element shows the correlation coefficient between subjects.

### 2.6 Network analysis

We extracted the RSNs identified in a previous study using independent component analysis (ICA) (Rao et al., 2021). ICA decomposes 4D functional MRI data (containing spatial and temporal information) into distinct spatial and temporal components. The purpose of ICA is to extract different brain networks by decomposing them in a way that ensures statistical independence between components. Specifically, the independence of spatial and temporal components is maximized, which enables the extraction of brain network components such as RSNs as significant independent components (Beckmann et al., 2005). The RSNs of default mode network (DMN), sensorimotor network (SMN), salience network (SN), and visuospatial network (VSN) were identified through ICA in this study. The DMN is primarily located in the posterior medial cortex, superior parietal lobule, parieto-occipital area, and visual area. The SMN is a local network of the sensorimotor cortices. The SN is a connectivity in the anterior cingulate cortex, prefrontal cortex and caudate nuclei. The VSN mainly encompasses the parieto-occipital area and visual cortices. We used the FMRIB Software Library (FSL) Multivariate Exploratory Linear Optimized Decomposition into Independent Components (MELODIC) to perform probabilistic group ICA (Smith et al., 2004). The multi-session temporal ICA concatenation approach was applied for all macaques in a temporally concatenated manner for ICA analysis. A total of 70 independent components were extracted from all macaques, and RSNs were extracted using the criteria of selecting voxels with Z-values greater than three from the outputted probabilistic spatial maps. The criterion was empirically determined within the range of Z-scores greater than 2 (Mueller et al., 2014; Yacoub et al., 2020). Then, we selected the RSNs corresponding to DMN, SMN, SN, and VSN, which matched the locations described above. The RSNs obtained from ICA were then used as ROIs for the calculation of FC, SC, ALFF, global correlation, and local correlation in all individuals using the CONN toolbox. FC was calculated as the Pearson’s correlation coefficient between the time course of fMRI signals within the RSNs. The ALFF was further calculated as the square root of the power spectrum in the frequency range of 0.008–0.09 Hz within each RSN (Yang et al., 2007). Global and local correlation (kernel size is 25 mm) involve calculating the average of correlation coefficients for each voxel in the brain. Global correlation represents the correlation of the averaged time course in the RSN with all other voxels (Saad et al., 2013), while local correlation focuses on the correlation of the averaged time course in the RSN with voxels neighboring the RSN (Deshpande et al., 2009).

### 2.7 Statistics analysis

The Pearson’s correlation coefficients and statistical significance (*p* < 0.05) between age and SC, FC, ALFF, and global and local correlation were calculated using “corr” function in MATLAB.

## 3 Results

### 3.1 Individual similarity of SC and FC

**Figure 1:**
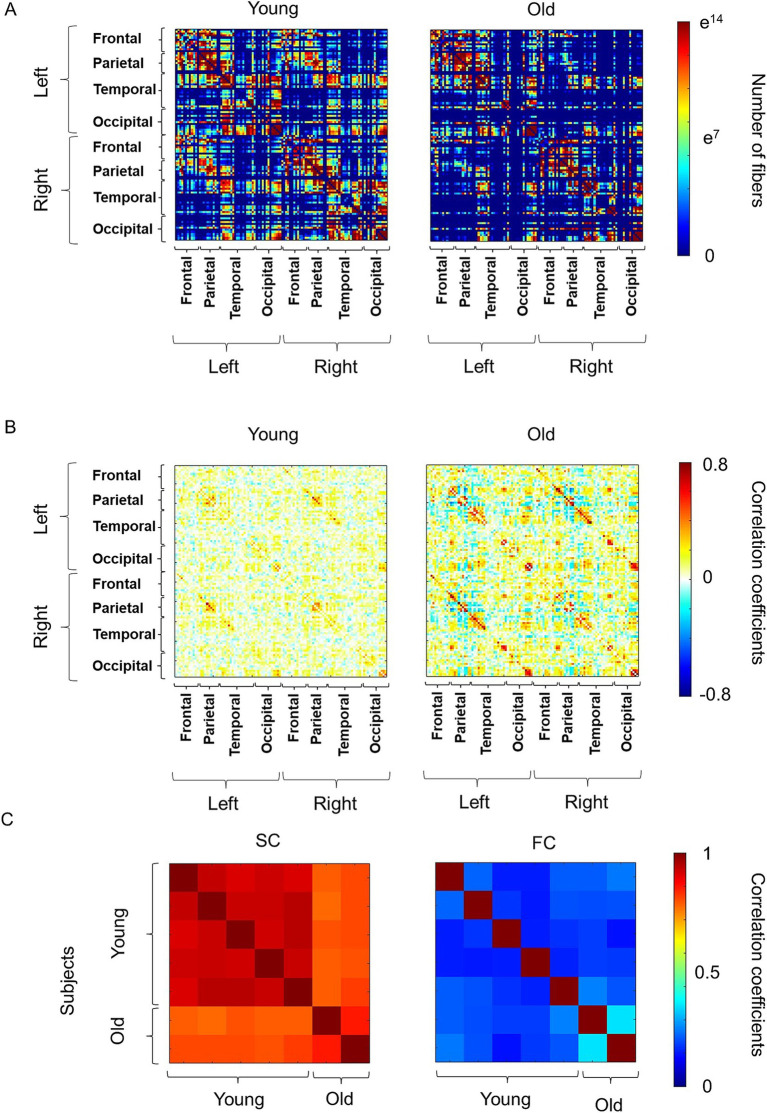
Overview of the connectivity matrix and similarity matrix. **(A)** The averaged structural connectivity (SC) in young and old macaques. The SC matrix is a symmetric matrix where each row and column correspond to region of interest (ROI), and each element indicates the neural fibers connecting each pair of ROIs. The color bar represents the number of fibers between the ROIs and “e” indicates Napier’s constant. **(B)** The averaged functional connectivity (FC) in young and old macaques. FC matrix is a symmetric matrix where each row and column correspond to an ROI, and each component of the FC matrix was displayed as a Fisher-transformed bivariate correlation coefficient between the BOLD time series of a pair of ROIs. The color bar represents the value of Pearson’s correlation coefficients between the regions of interest. **(C)** Individual similarity of SC and FC in macaques.

The averaged SC and FC of five young and two old macaques are shown in Figure 1. Statistical comparisons were not performed, because the sample size of the old macaques was too small (*n* = 2). The interhemispheric connections of the SC, which was assigned as the absolute number of streamlines connecting each pair of ROIs, appeared to be reduced in the old group (Figure 1A). In particular, the connections between the left and right frontal and parietal lobes and the connection with some regions of the temporal lobe seemed to be reduced in the old group. In contrast, the FC, which is the temporal correlation of the BOLD signal time-series data between each ROI, was similar between the young and old groups (Figure 1B). We subsequently assessed the inter-individual correlation coefficients as measures of similarity in SC and FC, respectively (Figure 1C). SC indicated high similarity within young macaques (*r* = 0.91–0.94, All *p*-values are below 0.01.), while old macaques show low similarity with young macaques (*r* = 0.77–0.82, All *p*-values are below 0.01.). The similarity of the FC was low among all subject, but with higher similarity between older macaques (*r* = 0.35, *p* < 0.01) than others (*r* = 0.11–0.25, All *p*-values are below 0.01.).

### 3.2 Age related changes in SC and FC

**Figure 2:**
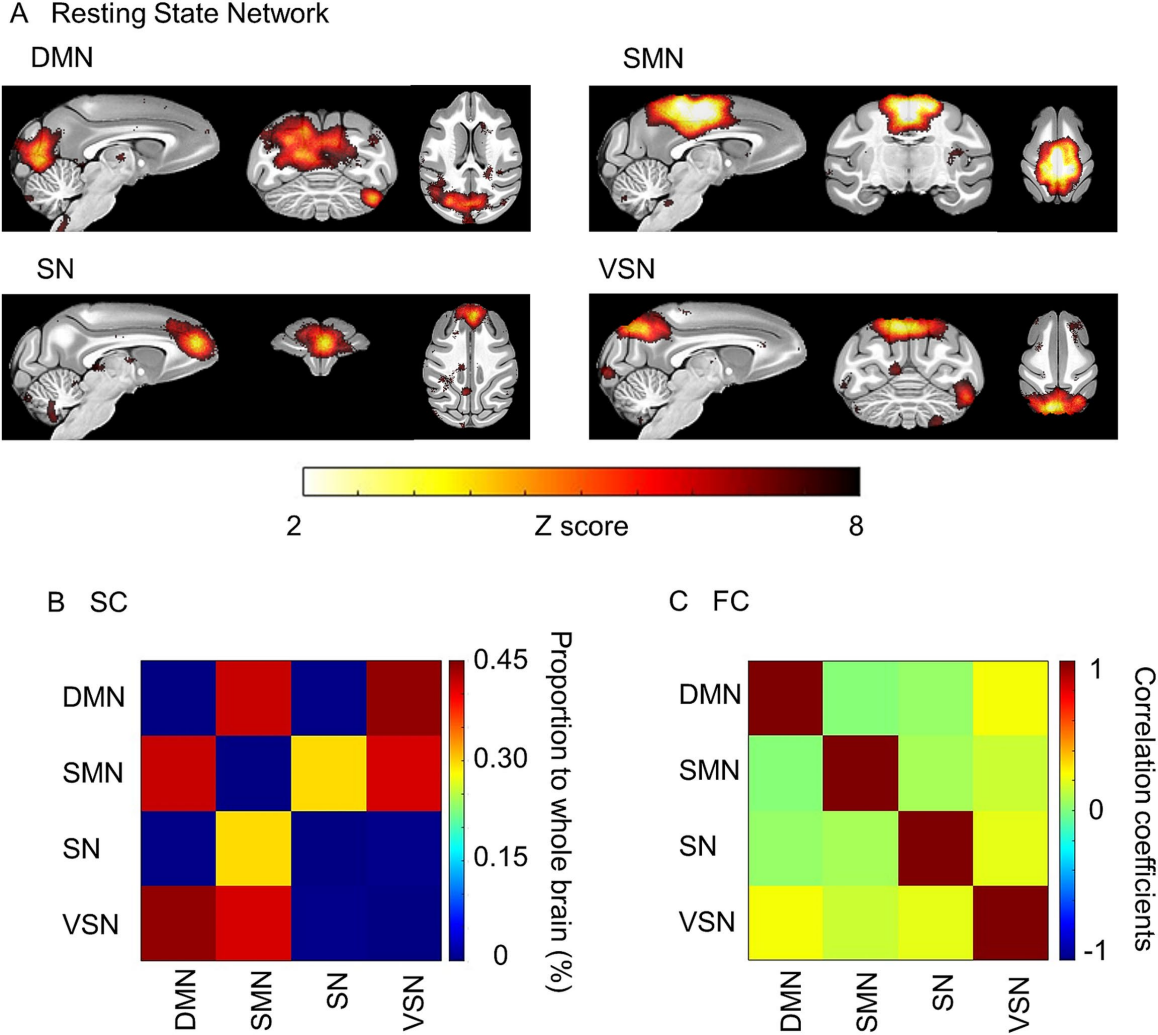
Resting state networks (RSN) extracted by independent component analysis. **(A)** ROIs of the Default mode (DMN), Sensorimotor (SMN), Salience (SM), and Visuospatial (VSN) networks were extracted. The color bar represents the value of the Z score. **(B)** The averaged structural connectivity matrix. The color bar represents the percentage of SC to the whole brain. **(C)** The averaged functional connectivity matrix between RSNs. The color bar represents Pearson’s correlation coefficient.

**Figure 3:**
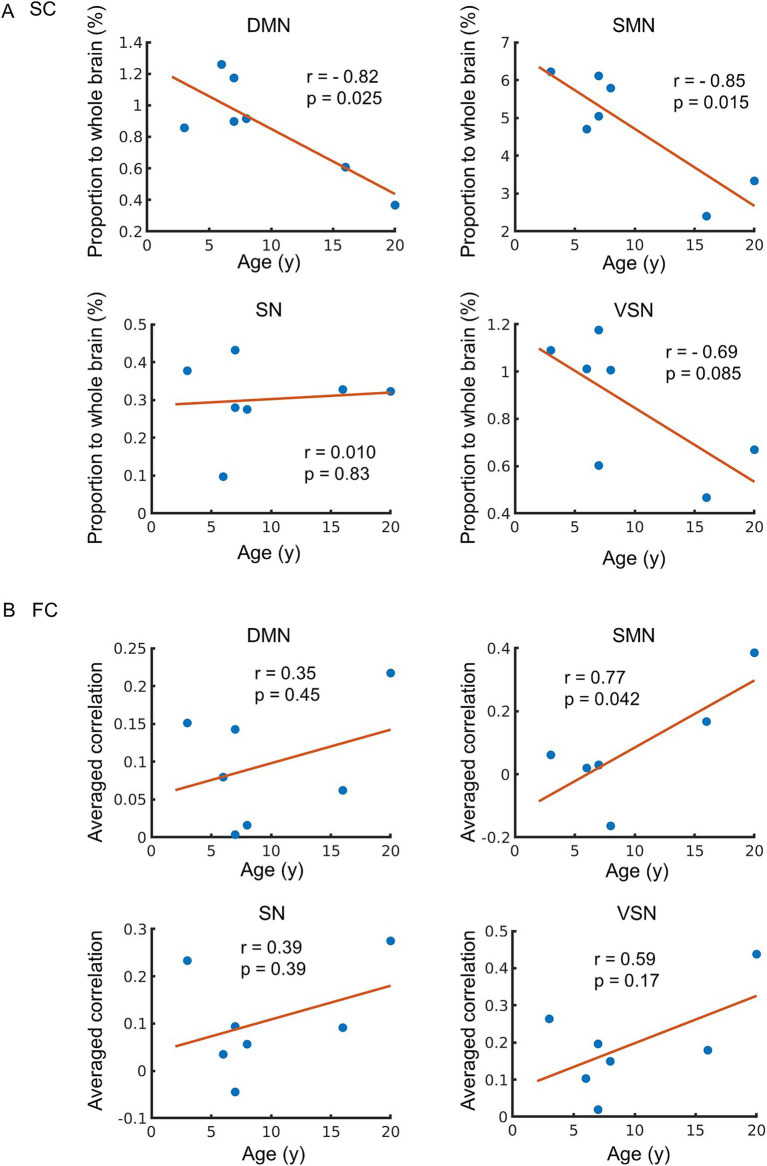
Age-related changes in structural connectivity (SC) and functional connectivity (FC). **(A)** The relationship between SC and age. The vertical axis of the figure represents the proportion of SC connected to each network relative to the whole brain, while the horizontal axis displays age. **(B)** The relationship between FC and age. The vertical axis of the figure represents the mean value of correlation between each network and other region, while the horizontal axis displays age. The regression line on the figure was constructed based on the observed data, and its slope is related to the correlation coefficient (*r*). The *p*-value indicates the significance of the relationship between the observed data and the line.

We analyzed the correlation between the functional and structural parameters of typical RSNs and age. RSNs extracted from all macaques were similar to the previous study (Rao et al., 2021) (Figure 2A). The proportions of SC and the average FC were calculated using these RSNs (Figures 2B,C). The SC of DMN-SMN, DMN-VSN, and SMN-VSN showed relatively higher proportion to SC in whole brain compared to other connections (Figure 2B). The FC of DMN-VSN showed relatively higher correlation coefficients than other connections (Figure 2C). Remarkably, significant negative correlations of SCs with age (*r* = −0.82) were found in DMN and SMN (*p* = 0.025 and 0.015, respectively) (Figure 3A). Although the correlation between SC and age in the VSN was not statistically significant (*p* = 0.085 > 0.05), a negative correlation (*r* = −0.69) was nevertheless observed. No significant correlation between SC and age was observed for the SN (*r* = 0.010, *p* = 0.83). In contrast, no correlation (*r* = 0.01) was observed between the SC and age in the SN. In contrast to the negative correlation between SC and age, FC was positively correlated with all RSNs (Figure 3B). Only the FC within the SMN showed a significant positive correlation with age (*r* = 0.77, *p* = 0.042) (Figure 3B). A positive correlation between age and FC in the DMN, SN, and VSN was also observed; however, the relationship was not significant (*r* = 0.35, 0.39, and 0.59, *p* = 0.45, 0.39, and 0.17, respectively).

### 3.3 Age related changes in ALFF, global correlation, and local correlation

**Figure 4:**
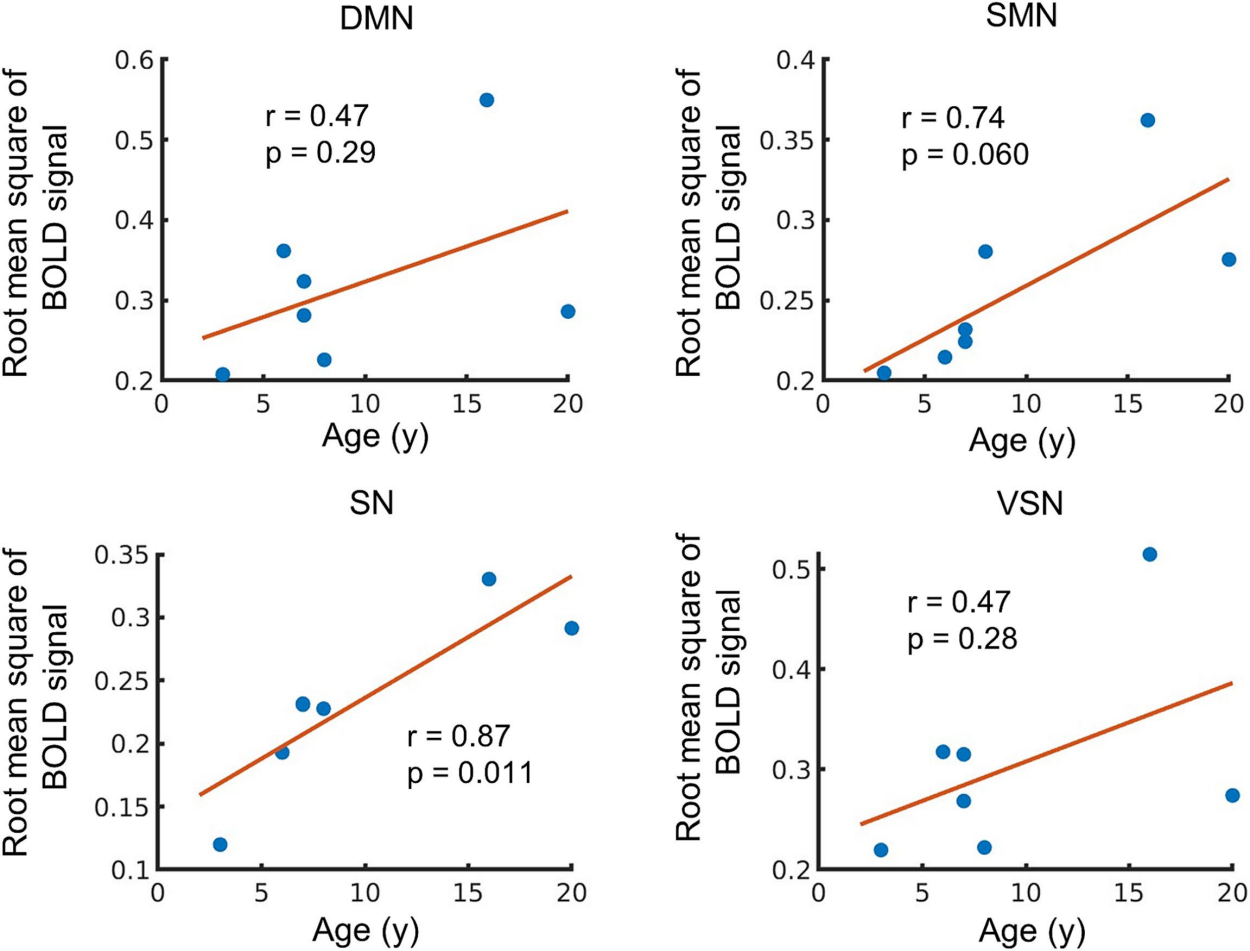
Age-related changes in amplitude low frequency fluctuations (ALFF). The vertical axis of the figure represents the averaged ALFF in each network, while the horizontal axis displays age. The regression line on the figure based on the observed data, and its slope is related to the correlation coefficient (*r*). The *p*-value indicates the significance of the relationship between the observed data and the line.

We investigated the ALFF within each RSN, because the local power of the low-frequency band-passed BOLD signal fluctuation reflects the neuronal activity in the resting state (Nakamura et al., 2020; Tsurugizawa et al., 2019). Overall, we observed a positive correlation with age in ALFF of all RSNs (*r* = 0.47 to 0.87), and only ALFF in SN was significantly correlated with age (*p* = 0.011) (Figure 4). The ALFF in the SMN was positively correlated with age (*r* = 0.74), but the correlation was not significant (*p* = 0.060). The ALFF in the DMN and VSN showed moderate correlations (DMN: *r* = o.47, *p* = 0.29; VSN: *r* = 0.47, *p* = 0.28). These results indicate the potential trend in the relationship between ALFF and age. However, the findings also suggest instability in the results due to the small sample size and the limited number of older monkeys.

**Figure 5:**
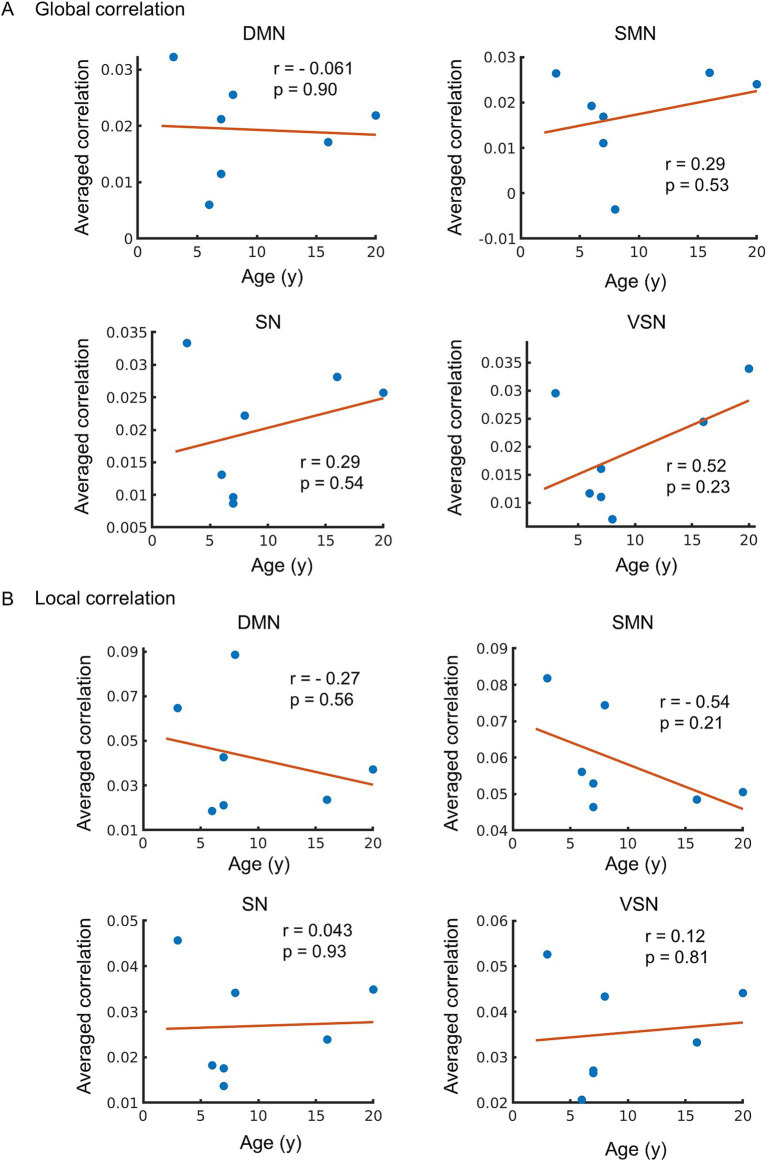
**(A)** The relationship between global correlation and age. The vertical axis of the figure represents the averaged value of global correlation in each network, while the horizontal axis displays age. The regression line on the figure was constructed based on the observed data, and its slope is related to the correlation coefficient (*r*). **(B)** Age-related changes in local correlation. The relationship between local correlation and age. The vertical axis of the figure represents the averaged value of local correlation, while the horizontal axis displays age. The regression line on the figure based on the observed data, and its slope is related to the correlation coefficient (*r*). The *p*-value indicates the significance of the relationship between the observed data and the line.

The correlations between the global and local correlation for each RSN were also investigated. A positive correlation between global correlation and age was observed in the SMN, SN, and VSN, but the correlation was weak and not significant (*r* = 0.29, 0.29, and 0.52, *p* = 0.53, 0.54, and 0.23, respectively) (Figure 5A). No correlation in global correlation in DMN with age was observed (*r* = −0.061 and *p* = 0.90). A moderate negative correlation in local correlation in SMN with age was observed, but did not reach significance (*r* = −0.54 and *p* = 0.21) (Figure 5B). A weak negative correlation was observed in the DMN (*r* = −0.27, *p* = 0.56), while no correlation was observed in the SN or VSN (*r* = 0.043 and 0.12, *p* = 0.93 and 0.81, respectively).

## 4 Discussion

In the present study, we demonstrated the age-related changes in brain function and structure within the typical RSNs of macaques. A negative correlation between age and SC was identified in specific RSNs, whereas FC and ALFF showed positive correlations with age. As FC and ALFF are caused by neuronal oscillation and activity, these differences could possibly reflect the age-related alterations in the two modalities, such as neuronal activity and brain structure.

### 4.1 Individual similarity of SC and FC

Aged macaques tended to have stronger local connection in the SC than young macaques (Figure 1). The similarity of the SC among macaques aged 5 to 9 years was high (*r* = 0.87–0.96) (Ouchi et al., 2024), and the results of this study showed similar findings among young macaques (*r* = 0.91–0.94). In contrast, the similarity between young and old macaques was relatively low (*r* = 0.77–0.82) (Figure 1C). Previous studies, which reported that the integrity of the SC decreases with age (Gong et al., 2009), may explain why the similarity in SC among young and old macaques was low (*r* = 0.77–0.82). Interestingly, the SC between the two old macaques showed higher similarity (*r* = 0.85) than that between old and young macaques, indicating that structural similarity is still conserved at each life stage.

The overall similarity in FC among young macaques and between young and old macaques was low (*r* = 0.11–0.25), possibly because anesthesia affects FC (Barttfeld et al., 2015; Hutchison et al., 2015; Tsurugizawa and Yoshimaru, 2021; Xu et al., 2019) but not SC. Importantly, although the FC strength is affected by anesthesia, some RSNs are preserved (Tsurugizawa and Yoshimaru, 2021). We successfully achieved typical RSNs, including the DMN, SMN, SN, and VSN, under anesthesia, consistent with a previous study (Rao et al., 2021). Regarding the similarity between old macaques, one prior study hypothesized that age-related reductions in integrity of the SC decrease variation in the FC, indicating that resting-state brain activity may depend on specific connections (Zhang et al., 2024). The higher similarity between old macaques (*r* = 0.35) than between young and old macaques (*r* = 0.11–0.25) may therefore be due to the reduction in the integrity of the SC. A previous study in humans also revealed that the global integration of the FC increases with age (Damoiseaux, 2017). However, the present study only observed similarities between the two old macaques. Further investigation using a larger sample size of older macaques is required for further analysis in future studies, although it is difficult to obtain aged macaques.

### 4.2 Age-related changes in RSNs

Here, we focused on four typical networks, namely the DMN, SMN, SN, and VSN, as these RSNs reflect the basal brain networks in the resting state, including in humans. The proportion of SC connected to the DMN, SMN, and VSN in the whole brain decreased with age; however, the percentage of SCs connected to the SN did not change (Figure 3A). Most SCs, including those in the hub regions of the DMN, decline in older subjects in humans (Zimmermann et al., 2016). The DMN is characterized as being active during wakeful rest compared to when performing a cognitive task in humans and macaques. A previous human study showed that the SC in the SN is less affected by aging (Damoiseaux, 2017). The ventral SN is related to affective experiences, whereas the dorsal SN is associated with attention and processing speed (Touroutoglou et al., 2012). Another study reported that the ventral SN is homologous between humans and macaques (Touroutoglou et al., 2016). These results are consistent with those of the current study. A significant negative correlation was observed between DMN and SMN, but not between SN and VSN. For SN, we hypothesized that this was due to its resistance to age-related decline. Regarding VSN, although a trend toward negative correlation was observed, the lack of statistical significance can be attributed to limited statistical power due to the small sample size.

In contrast to SC, FC was positively correlated with all RSNs (Figure 3B). A previous study in humans indicated that FC with the DMN in old subjects, defined as those aged 60–83 years old, was greater than that in younger individuals (Grady et al., 2016; Spreng et al., 2016). Another study that investigated FC with across more classified ages revealed that although alterations in FC are not proportional to age, the FC strength and density in the DMN and SMN in over 80 years old group were greater than those in individuals of less than 60 years old (Farras-Permanyer et al., 2019). These results were consistent with the current study. However, it indicates that disparities in the definition of “old” are not strictly decided and the definition of “old” macaques may affect the results of altered FC. This study therefore investigated the correlation of FC with age instead comparing between “young” and “old,” as in previous studies.

We further observed a positive correlation between the ALFF and age across all RSNs, indicating an increase in the power of the low-frequency components of the MRI signal in old macaques. Since ALFF is considered a biomarker reflecting neural activity in the resting state (Nakamura et al., 2020; Tsurugizawa et al., 2019; Yang et al., 2007), the positive correlation of ALFF within the SMN and SN with age indicates higher resting-state neural activity in older macaques than in young macaques. A previous human study reported that ALFF increases with age in humans (Zhang et al., 2021). Furthermore, an increase in the ALFF is correlated with delayed attention/processing speed in the left cerebellar posterior lobe and left superior parietal gyrus, providing evidence that longitudinal cognitive decline shows a frequency-specific correlation with ALFF changes (Fan et al., 2022). Since ALFF reflects neural activity (Chang et al., 2005), the age-related increase in action potential firing rates may be associated with higher ALFF observed in older macaques. As such, the age-related changes in ALFF observed in this study suggest that they may be associated with cognitive decline in macaques. However, it has been reported that anesthesia increases the ALFF in the bilateral prefrontal cortex of macaques (Lv et al., 2016), and the influence of physiological changes such as respiration and heart rate need to be considered.

Prior studies have shown that local correlation decreases with age and is correlated with language abilities in humans (Pistono et al., 2021). Additionally, in motor networks, performance has been reported to correlate with local correlation (Wu et al., 2007). Thus, the results of this study showing that the local correlation in the DMN and SMN decreased with age (Figure 5) may indicate a factor explaining behavioral differences, although it was not statistically significant. In contrast, no correlation with age was observed in the SN or VSN, suggesting that the degree of age-related impact may vary depending on the network. Graph theory studies have demonstrated that local efficiency in resting-state brain activity measured using fMRI decreases with age, whereas global efficiency does not change significantly (Cao et al., 2014; Geerligs et al., 2015). Furthermore, local correlation have been reported to explain glucose metabolism in the brain, which decreases with age (Bernier et al., 2017; Subtirelu et al., 2023). The present study showed that SC and local correlation within the SMN were negatively correlated with age (Figures 3–5), which is in agreement with the results of a previous study that showed decreased white matter integrity (Gong et al., 2009) and glucose metabolic rate (Subtirelu et al., 2023) in SMN with aging. In contrast, there is a positive correlation between FC and ALFF in all RSNs with age-compensatory spontaneous neural activity increases (Spooner et al., 2019). Due to low individual similarity in functional MRI data (Figure 1C), we acknowledge that the small number of older monkeys may have led to unstable results. Given the small sample size and the potential for outliers, the age-related trends observed in the data should be interpreted cautiously. This is considered to be the factor responsible for both the RSNs that showed significant correlations and those that did not show significant correlations.

## 5 Conclusion

Typical RSNs such as the DMN of non-human primates are homologous to those in humans; however, MRI study on the age-related changes in FC and SC in macaques are much less common than human studies. The current study showed age-related changes in the structure and resting-state function of typical RSNs in macaques, which are similar to those in humans as described above. These results indicate the possibility that aging study in macaques can contribute to the effect of aging on the functional network in humans.

## Data Availability

The raw data supporting the conclusions of this article will be made available by the authors, without undue reservation.

## Footnotes

^1^ https://www.jnss.org/en/animal_primates

^2^ https://fsl.fmrib.ox.ac.uk/fsl/
